# Detection of alpha- and betacoronaviruses in rodents from Yunnan, China

**DOI:** 10.1186/s12985-017-0766-9

**Published:** 2017-05-26

**Authors:** Xing-Yi Ge, Wei-Hong Yang, Ji-Hua Zhou, Bei Li, Wei Zhang, Zheng-Li Shi, Yun-Zhi Zhang

**Affiliations:** 1 Key Laboratory of Special Pathogens and Biosafety, Wuhan Institute of Virology, Chinese Academy of Sciences, Wuhan, 430071 China; 2 Yunnan Provincial Key Laboratory for Zoonosis Control and Prevention, Yunnan Institute of Endemic Diseases Control and Prevention, Dali, 671000 China; 3 School of Public Health, Dali University, Dali, 671000 China; 4 College of Biology, Hunan University, Changsha, 410082 China

**Keywords:** Rodent, Coronavirus, Genetic diversity

## Abstract

**Background:**

Rodents represent the most diverse mammals on the planet and are important reservoirs of human pathogens. Coronaviruses infect various animals, but to date, relatively few coronaviruses have been identified in rodents worldwide. The evolution and ecology of coronaviruses in rodent have not been fully investigated.

**Results:**

In this study, we collected 177 intestinal samples from thress species of rodents in Jianchuan County, Yunnan Province, China. Alphacoronavirus and betacoronavirus were detected in 23 rodent samples from three species, namely *Apodemus chevrieri* (21/98), *Eothenomys fidelis* (1/62), and *Apodemus ilex* (1/17). We further characterized the full-length genome of an alphacoronavirus from the *A. chevrieri* rat and named it as AcCoV-JC34. The AcCoV-JC34 genome was 27,649 nucleotides long and showed a structure similar to the HKU2 bat coronavirus. Comparing the normal transcription regulatory sequence (TRS), 3 variant TRS sequences upstream the spike (S), ORF3, and ORF8 genes were found in the genome of AcCoV-JC34. In the conserved replicase domains, AcCoV-JC34 was most closely related to *Rattus norvegicus* coronavirus LNRV but diverged from other alphacoronaviruses, indicating that AcCoV-JC34 and LNRV may represent a novel alphacoronavirus species. However, the S and nucleocapsid proteins showed low similarity to those of LRNV, with 66.5 and 77.4% identities, respectively. Phylogenetic analysis revealed that the S genes of AcCoV-JC34, LRNV, and HKU2 formed a distinct lineage with all known coronaviruses.

**Conclusions:**

Both alphacoronaviruses and betacoronaviruses were detected in *Apodemus chevrieri* in the Yunnan Province of China, indicating that *Apodemus chevrieri* is an important host for coronavirus. Several new features were identified in the genome of an *Apodemus chevrieri* coronavirus. The phylogenetic distance to other coronaviruses suggests a variable origin and evolutionary route of the S genes of AcCoV-JC34, LRNV, and HKU2. These results indicate that the diversity of rodent coronaviruses is much higher than previously expected. Further surveillance and functional studies of these coronaviruses will help to better understand the importance of rodent as host for coronaviruses.

**Electronic supplementary material:**

The online version of this article (doi:10.1186/s12985-017-0766-9) contains supplementary material, which is available to authorized users.

## Background

Coronaviruses (CoVs) are enveloped viruses in the *Coronaviridae* family that contain a positive-sense and single-stranded RNA genome of approximately 30 kilobases [1]. CoVs consist of 4 genera and have been identified in a wide range of animals and in humans. Members of the *Alphacoronavirus* (α-CoV) and *Betacoronavirus* (β-CoV) infect mammals, and members of the *Gammacoronavirus* (γ-CoV) and *Deltacoronavirus* (δ-CoV) mainly infect avian species [2–4]. As important etiological agents, CoVs have been recognized in human and animals and cause upper respiratory diseases in most cases. To date, 6 human CoVs were discovered: 4 of them (HCoV-229E, NL63, OC43, and HKU1) mainly cause mild respiratory diseases, and the other 2, severe acute respiratory syndrome coronavirus (SARS-CoV) and Middle East respiratory syndrome coronavirus (MERS-CoV) cause severe respiratory diseases [5, 6].

The SARS-CoV outbreak boosted the discovery of novel CoVs in various animals, particularly in bats. Over 140 novel bat coronaviruses (species or genotypes) have been discovered since the SARS outbreak [7, 8]. Furthermore, there is strong evidence to show that SARS-CoV, MERS-CoV, and HCoV229E may have evolved from bat CoVs [9–13].

Rodents are the most diverse mammals on the planet and have been documented as important carriers of human diseases [14]. Although murine hepatitis virus (MHV) has been used as a model to study CoV for a long time, limited information is available regarding the prevalence and diversity of rodent CoVs [15–18]. Recently, several novel α-CoVs and β-CoVs (LRNV, LAMV, LRLV, and HKU24) were identified in rodents in China and Europe [19–21]. These discoveries suggested that rodents may carry diverse, unrecognized CoVs [22]. In the present study, we describe the first discovery of CoVs in 3 different rodent species in the Yunnan Province of China and report a much higher (21.4%) detection rate of CoV nucleic acid in *A. chevrieri* than in other rodent species studied previously (<5%) [19, 20]. In addition, this is the first report of finding α-CoV and β-CoV in the same rodent species in China.

## Methods

### Sample collection

In October 2011, for pest control and routine pathogen surveillance, 177 rodents were captured in the bush and grass near the cropland ridge in Jianchuan County of the Yunnan Province (Additional file 1: Figure S1). Animal intestines were collected and transferred to liquid nitrogen for temporary preservation and transport. Following arrival at the laboratory, the samples were stored at –80 °C until they were used for virus detection. Animal species were first identified based on morphology and further by DNA sequencing of the mitochondrial cytochrome b (*CytB*) gene with ready-to-use methods [23].

### RNA extraction

To extract viral RNA, 50 mg of intestinal tissue samples were homogenized using 1 ml PBS. Homogenates were centrifuged and RNA was extracted from 140 μL supernatant using the QIAamp Viral RNA Mini Kit (Qiagen) following the manufacturer’s instructions. Extracted RNA was used as a template for amplifying the mitochondrial *CytB* gene with the primers *CytB*F (5′-ATGATATGAAAAACCATCGTTG-3′) and *CytB*R (5′-TTTCCNTTTCTGGTTTACAAGAC-3′). The 1.2-kb replicons were gel purified (Promega, Madison, USA) and directly sequenced using the forward and reverse primers with a 3100 Sequencer (Applied Biosystems, Waltham, MA, USA).

### Reverse transcription PCR (RT-PCR) screening of CoVs

The 440-bp RNA-dependent RNA polymerase gene (RdRp) fragment of CoVs was amplified by previously described methods using a One-Step RT-PCR (Invitrogen, San Diego, USA) [24]. PCR target bands were gel purified and sequenced on a 3100 Sequencer (ABI, Waltham, MA, USA). Standard precautions were taken to avoid PCR contamination, and no false positives were observed in the negative controls. To determine the heterogeneity of the amplicons, we inspected the sequencing chromatograms. No overlapping multicolor peaks were found, indicating that no CoV co-infection occurred in the animals examined in this study. To confirm the PCR results, positive samples were verified by performing two independent PCRs. The CoV-positive samples were named using the rodent species name, the location (Jianchuan County), and the sample number. For example, a CoV detected in *A. chevrieri* (sample number 54) was named as *A. chevrieri* CoV JC54 (AcCoV-JC54).

### Viral culture

Three positive rodent samples representing different CoVs (JC30, β-CoV; JC34 and JC54, α-CoV) were used to perform viral isolation in Vero E6 cells (African green monkey kidney cells, ATCC: CRL-1586).

### Genome sequencing

To sequence the viral genome, 140 μL supernatant from a JC34 tissue homogenate was treated using viral metagenomics procedures and ready-to-use methods [25]. Synthesized DNA was used to construct the sequencing library, and next-generation sequencing (*NGS*) was performed using an HiSeq-PE150 instrument (Illumina/Solexa). BLAST searches were performed against the CoV database, and 413,599 reads homologous to CoV sequences were found. The reads were processed using Geneious (Version 5.5.9, Biomatters Limited, Auckland, New Zealand) to assemble a near full-length CoV genome contig. Subsequently, 5′ and 3′ RACE (Takara) were performed to confirm the ends of the genome sequence using two primers (5′-CAGGACGTCTAATGCAATACCT-3′ and 5′-AACACACTGAAATCAGACCTTG-3′), which were designed based on the obtained contig sequences and primers supplied in the kits. The replicons were both end sequenced. Finally, all sequences were assembled with the CoV contig to obtain a full-length CoV genome, designated as AcCoV-JC34.

### Sequence analysis

The genome sequence of AcCoV-JC34 was compared to other representative CoVs with complete genomes available. The open reading frames (ORFs), deduced amino acid sequences, and potential cleavage sites in orf1ab were predicted by ORF Finder (NCBI) and ZCURVE_CoV 2.0 [26]. Sequence alignment and editing were performed using ClustalW (Version 2.0), BioEdit (Version 7.1.9), and Geneious (Version 5.5.9) [27, 28]. The spike (S) protein structure of AcCoV-JC34 was searched against sequences in the Protein Data Bank (PDB) and predicted using a web-based SWISS-MODEL server. The cleavage sites in the S protein were predicted by comparing amino acid sequences, combined with analysis using a web-based ProP server [29]. Phylogenetic trees were constructed using the maximum-likelihood (ML) algorithm, with bootstrap values determined by 1000 replicates in MEGA6 and PhyML software [30, 31].

## Results

### Detection of α-CoVs and β-CoVs in rodents

A total of 177 intestinal samples were obtained from rodents, including three different species. By RT-PCR, targeting a 440 base pairs (bp) partial RdRp gene sequence of CoVs, 23 rodents were identified as CoV positive, which included 21 of 98 (21.4%) *A. chevrieri*, 1 of 17 (5.9%) *A. ilex*, and 1 of 62 (1.6%) *E. fidelis* samples (Table 1). The obtained *CytB* gene sequences were deposited in GenBank under accession numbers KX964655–KX964657. The isolation of rodent CoV from VeroE6 cells was not successful.

**Table 1:** Detection of coronavirus in rodents by RT-PCR

Scientific name	No. of rodents tested	No. of positives for the following/total no. of animals tested (%):	Detected coronaviruses
*Apodemus chevrieri*	98	21/98 (21.4%)	*Alphacoronavirus* and *Betacoronavirus*
*Apodemus ilex*	17	1/17 (5.9%)	*Betacoronavirus*
*Eothenomys fidelis*	62	1/62 (1.6%)	*Alphacoronavirus*
*Total*	177	23/177 (13%)	

**Fig. 1:**
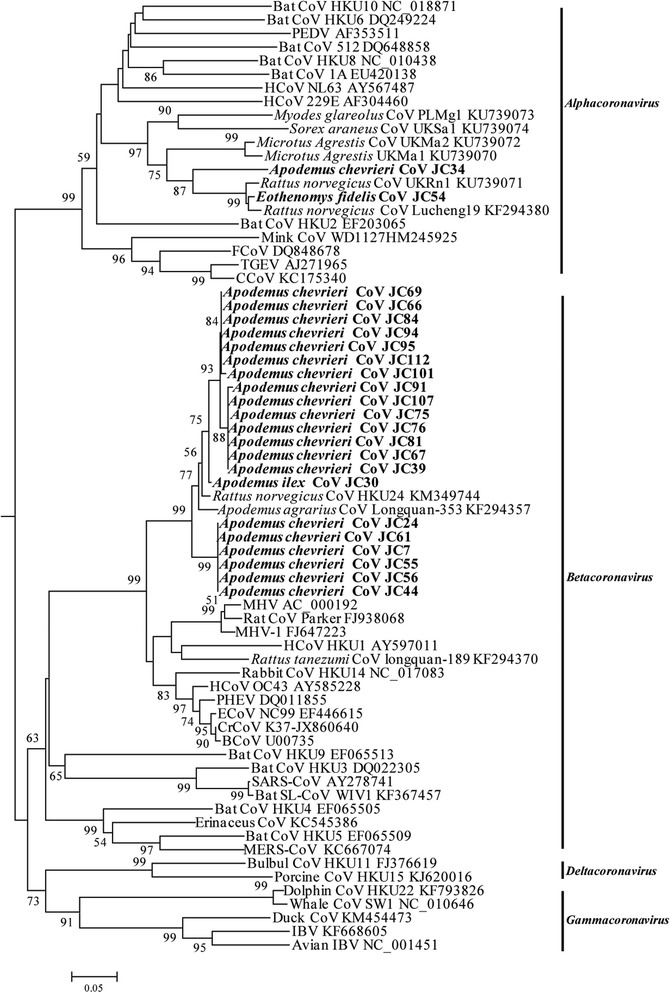
Phylogenetic analysis of detected rodent CoVs with representative CoVs based on 440-bp partial RdRp sequences. The tree was constructed by the maximum-likelihood method with 1000 bootstrap replicates. Bootstrap values above 50% were shown. Rodent CoVs found in this study are shown in bold. CoV abbreviations: bat SL-CoV WIV1, bat SARS-like coronavirus WIV1; BCoV, bovine coronavirus; CCoV, canine coronavirus; CrCoV, canine respiratory coronavirus; ECoV, equine coronavirus; FCoV, feline coronavirus; HCoV 229E, human coronavirus 229E; HCoV HKU1, human coronavirus HKU1; HCoV NL63, human coronavirus NL63; HCoV OC43, human coronavirus OC43; IBV, infectious bronchitis virus; MERS-CoV, MERS coronavirus; MHV, murine hepatitis virus; PHEV, porcine hemagglutinating encephalomyelitis virus; SARS-CoV, SARS coronavirus; TGEV, transmissible gastroenteritis virus

Partial RdRp sequences were searched against the CoV database, and the results indicated that 21 out of 23 sequences were β-CoVs, whereas the remaining two were α-CoVs. The β-CoV-related sequences had high nucleotide (nt) identities of 95–99% compared to the unclassified β-CoVs detected in rodents in China, Longquan-343 (*A. agrarius*) and HKU24 (*R. norvegicus*). The *A. chevrieri* CoV JC34 (AcCoV-JC34) and *E. fidelis* CoV JC54 (EfCoV-JC54) shared the highest nt identities of 84 and 98%, respectively, with the unclassified α-CoVs detected from *R. norvegicus*, LRNV (GenBank no: KF294380) in China or UKRn1 (GenBank no: KU739071) in Europe (Fig. 1). LRNV has a complete genome of 28,763 bp and UKRn1 (KU739071) has a partial RdRp sequence of 630 bp. Sequenced RdRp fragments in this study were deposited in GenBank under accession numbers: KX964650–KX964654.

### Genome organization and ORFs of AcCoV-JC34

One positive sample (JC34) was chosen for further sequencing to obtain the full-length genome because it showed low sequence similarity to other CoVs and appeared to be a novel CoV. By random PCR and Illumina sequencing, a near full-length genome of CoV was assembled from 413,599 reads. After sequencing 5′- and 3′-rapid amplification of cDNA end replicons, a complete genome was characterized. This virus was named rodent AcCoV-JC34 and the complete genome sequence was deposited in GenBank under accession number KX964649.

**Fig. 2:**
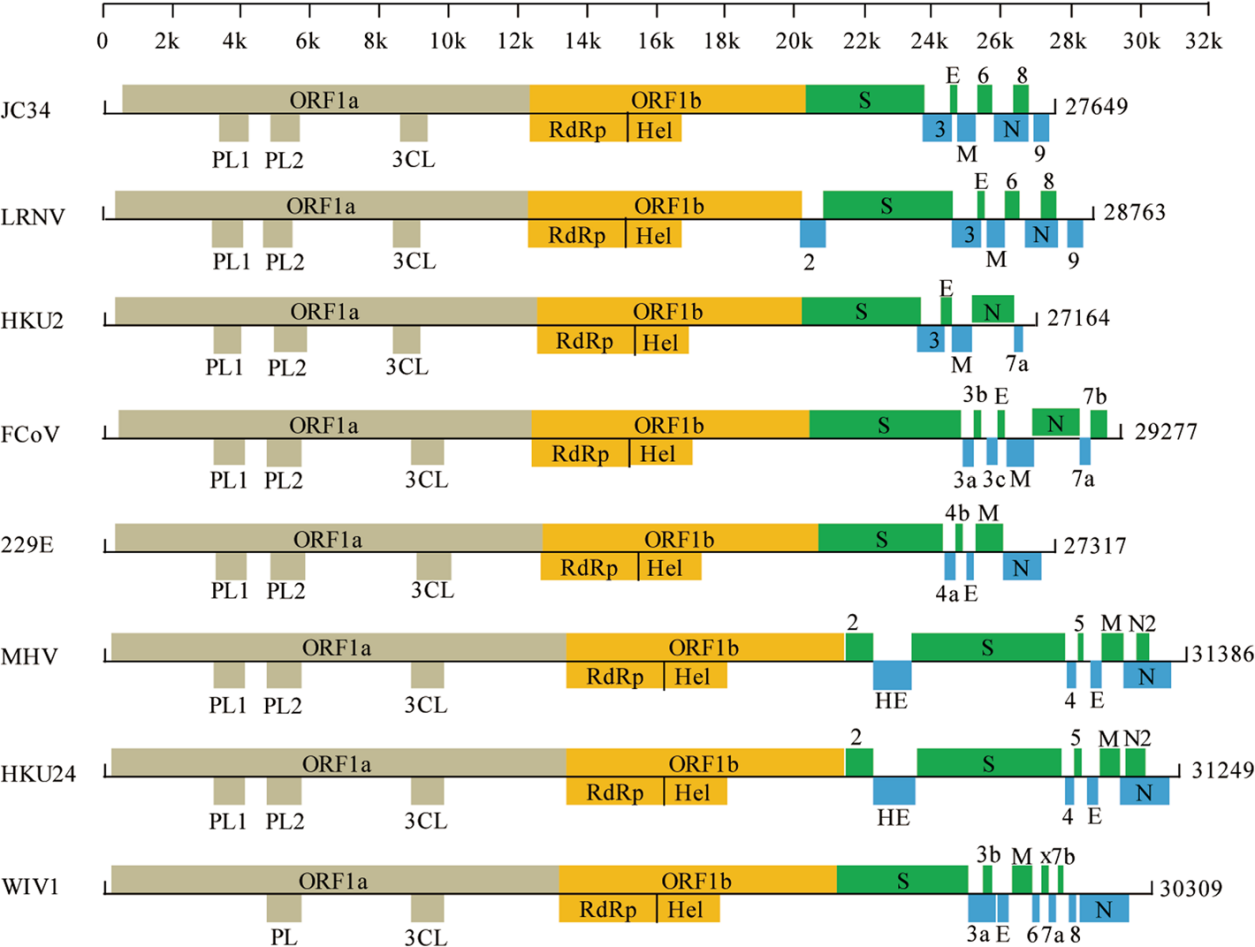
Comparison of the genome organizations of AcCoV-JC34, LRNV, HKU2, FCoV, 229E, MHV, HKU24, and WIV1. Predicted ORFs and 5 conserved domains are indicted by the boxes. Abbreviations: 3CL, 3C-like protease; E, envelope; HE, hemagglutinin-esterase; Hel, helicase; M, membrane; N, nucleocapsid; PL1pro and PL2P, papain-like proteases 1 and 2; RdRp, RNA-dependent RNA polymerase; S, spike

The genome size of AcCoV-JC34 was 27,649 bp and the G + C content was 40%. Similar to other α-CoVs, AcCoV-JC34 has a concise genomic organization and genes characteristic of CoV, including (from 5′ to 3′) the ORF1ab, S, envelope (E), membrane (M), and nucleocapsid (N) genes (Table 2 and Fig. 2). In addition, ORFs likely coding for accessory proteins ORF3, ORF6, ORF8, and ORF9 were also found.

**Table 2:** Gene similarities of AcCoV-JC34 and representative *Alpha-* and *Beta-*CoVs with full-length genome

Viruses	Genome		Pairwise amino acid sequence identity between JC34 and other virus sequences (%)						
	Size (nt)	G + C content(%)	3CL^pro^	RdRp	Hel	S	E	M	N
*Alphacoronavirus*									
RatCoV-JC34	27,649	40.07	-	-	-	-	-	-	-
BatCoV 1A	28,326	38.14	57	76.6	74	17.6	36.5	50.2	23.3
BatCoV HKU2	27,164	39.32	58.5	75.1	73.3	39.2	36	53.5	27.9
BatCoV HKU8	28,773	41.79	61.2	76.5	73.5	18.6	33.8	51	23.5
RatCoV LRNV#	28,763	40.29	91.9	94.3	96	67.2	93.8	92.3	78
BatCoV 512	28,203	40.12	60.9	73.3	71.8	18.3	35.5	53.1	24.6
PEDV	28,033	42.02	59.7	74.3	72	17	34.2	52.7	23.2
HCoV-NL63	27,553	34.46	59.8	73.9	73	17.2	35.1	50.8	24
HCoV-229E	27,317	34.46	58.9	73.3	74.3	19	36.4	46.3	21.7
MiCoV	28,941	37.47	59.7	77.9	73	17.5	40.7	53.2	25.9
FCoV	29,277	38.34	57.8	75.3	71.4	16.8	42	55.6	24.7
CCoV	29,380	37.56	58.1	76.4	71.9	16.9	40.7	57.3	25.6
TGEV	28,586	37.6	58.5	76.5	71.9	17.3	37	56.9	26.1
*Betacoronavirus* lineage A									
Betacoronavirus 1									
HCoV-OC43	30,741	36.69	44.1	55.8	59.1	21.8	17.6	37.4	21
PHEV	30,480	37.25	44.1	55.9	59.1	21.4	18.8	37.4	20.9
BCoV	31,032	37.02	44.4	55.7	59.5	22.1	18.8	38.7	21.4
ECoV	30,992	37.23	44.4	56	59.5	21.5	17.6	37.4	21.7
CrCoV	31,028	37.01	44.4	55.8	59.5	21.9	18.8	38.3	21.2
Murine coronavirus									
MHV	31,386	41.88	42.9	55.5	57.5	21.1	18.1	36.8	21.3
RCoV	31,250	41.26	42.9	56	58.9	21	20.5	38	21.3
RatCoV HKU24#	31,249	40.07	43.3	56	60.5	21.6	20.5	39.6	21
RatCoV-708#@	30,821	39.06	44.1	56	58.5	20.6	20.5	36.8	20.8
HCoV-HKU1	29,926	32.06	44.8	55.8	58.3	21.2	30	37.9	21.8
RabbitCoV HKU14	31,100	37.64	44.8	56	59.7	21.8	17.6	38.7	21.5
*Betacoronavirus* lineage B									
SARS-CoV	29,727	40.80	43	58.9	60.8	22.1	19	27	19.4
*Betacoronavirus* lineage C									
MERS-CoV	30,112	41.18	40.9	58.7	63.6	21.2	19.3	34.8	18.6
*Betacoronavirus* lineage D									
BatCoV HKU9	29,114	41.05	39.8	57.2	62.3	22.2	17.7	29.6	16

#: novel detected unclassified rat coronaviruses; @: a near complete genome

A hexanucleotide transcriptional regulatory sequence (TRS) is in the 5′-leader sequence and is required for the transcription of subgenomic RNAs, which is a unique characteristic of CoVs. Similar to rat CoV LRNV, bat CoV HKU2, and human CoV NL63, a putative TRS motif, 5′-AACUAA-3′ was found upstream of each ORF except for S, ORF3, and ORF8 in the AcCoV-JC34 genome. Instead of the 5′-AACUAA-3′ motif, the S, ORF3, and ORF8 genes had a variant TRS, 5′-AACUUA-3′, 5′-UACUAA-3′, and 5′-CACUAA-3′, respectively (Table 3).

**Table 3:** Locations of predicted ORFs in the genome of AcCoV-JC34

ORF	Location (nt)	Length (nt/aa)	TRS location	TRS sequence(s) (distance to AUG)
1ab	437–20376 (shift at 12361)	19940/6645	181	CAACTC**AACUAA**ACG(247)ATG
S	20376–23756	3381/1126	20306	TGGTA**AACUUA**TTGTTAG(64)ATG
3	23753–24397	645/214	23711	TGTTG**UACUAA**ACCT(36)ATG
E	24397–24633	237/78	24392	CCACTT**AACUA** **A** TG
M	24643–25389	747/248	24636	TTGATC**AACUAA**AATG
6	25401–25901	501/166	25389	GGTCTA**AACUAA**ACCATTATG
N	25903–27072	1170/389	25896	AATTTC**AACUAA**AATG
8	26530–27702	543/180	26484	TAGATCG**CACUAA**(40)ATG
9	27074/27391	318/105	27060	TGATGA**AACUAA**CGCCTAAAATG

Start codons are underlined. The conserved (AACUAA) or variant (AACUUA, UACUAA, CACUAA) TRS core sequences are highlighted in bold

Sixteen putative nonstructural proteins (nsp1 to nsp16) coded by ORF1ab of the AcCoV-JC34 were predicted (Table 4). The overall amino acid (aa) identity between the ORF1a and ORF1b polyproteins of AcCoV-JC34 and those of LRNV were 76 and 93.5%, respectively, but <60% relative to those of the other α-CoVs. The most conserved proteins 3CL^pro^ (nsp5), RdRp (nsp12), and Hel (nsp13) of AcCoV-JC34 possessed high aa identities, ranging from 91.9 to 96% compared to those of LRNV, but possessed low aa identities ranging from 57 to 77.9% compared to those of other known α-CoVs (Table 2). In addition, similar to the normal cleavage sites found in polyprotein of α-CoVs, 10 different cleavage sites were predicted between the nsps of AcCoV-JC34 (Table 4).

**Table 4:** Prediction of nsp1 to nsp16 and the cleavage sites of polyproteins 1a and 1b of the AcCoV-JC34

nsp	First-last amino acid residues	Protein size (aa)	Cleavage site	Putative functional domain(s)
nsp1	M^1^-G^218^	218	SCPCG|KSAFT	Unknown
nsp2	K^219^-G^820^	602	WVCKCG|AEVQLS	Unknown
nsp3	A^821^-A^2277^	1457	TRVGTA|DLAVFN	ADRP, PL1^pro^, PL2^pro^
nsp4	D^2278^-Q^2779^	502	LNAQ|SCAK	Hydrophobic domain
nsp5	S^2780^-Q^3042^	263	AKVQ|IEGA	3CL^pro^
nsp6	I^3043^-Q^3319^	277	SGMQ|CSWA	Hydrophobic domain
nsp7	C^3320^-Q^3437^	118	STIQ|SKLT	Unknown
nsp8	S^3438^-Q^3715^	278	VKLQ|NNEI	Unknown
nsp9	N^3716^-Q^3820^	105	VRLQ|AGKP	Unknown
nsp10	A^3821^-Q^3955^	135	STVQ|SNII	Unknown
nsp11	S^3956^-N^3974^	19	-	Short peptide at the end of ORF1a
nsp12	L^3956^-Q^4881^	926	SVLQ|SAGL	RdRp
nsp13	S^4882^-Q^5478^	597	TDLQ|SVLS	Hel
nsp14	S^5479^-Q^5998^	520	PILQ|GLEN	ExoN
nsp15	G^5999^-Q^6334^	346	PQLQ|NSEW	NendoU
nsp16	N^6345^-K^6646^	302	-	O-MT

Superscript numbers indicate positions in polyprotein pp1ab, with the supposition of a ribosomal frame shift resulting in a peptide bond between N^3974^/L^3975^ for the expression of ORF1ab

The S protein of AcCoV-JC34, consisting of 1126 amino acid residues, is predicted to be a type-I membrane glycoprotein with a signal peptide (residues 1 to 19), an extracellular region (residues 20 to 1070), a transmembrane domain (residues 1071 to 1093), and an intracellular region (residues 1094 to 1126) (Additional file 1: Figure S2). A fusion peptide (FP) and two heptad repeats (HR1 and HR2) important for membrane fusion and viral entry were located at residues 674 to 692 for FP, 753 to 840 for HR1, and 1029 to 1058 for HR2. The S protein of AcCoV-JC34 showed the highest aa similarity of 66.5% compared with rat CoV LRNV, followed by 39.2% compared with BtCoV HKU2. Proteolysis of the S protein plays a pivotal role in the activation of viral and cell membrane fusion. Two cleavage sites, one located at residue 508 at the S1/S2 interface (RRAR/AR), and the other located at residue 674 (R/S) at the S2′ position, were predicted by comparing aa sequences based on analysis with a web-based ProP program (Additional file 1: Figure S2). The S1 region of AcCoV-JC34 has an N-terminal domain (NTD) and C-terminal domain (CTD). Both the NTD and CTD showed low aa sequence identities of <25% with those of other very well characterized CoVs. One of them was responsible for receptor recognition and binding, but due to the high dissimilarity with known receptor-binding domains (RBDs), it was difficult to determine the precise location of the RBD of AcCoV-JC34.

The AcCoV-JC34 proteins ORF3, E, M, ORF6, N, ORF8, and ORF9 also had low aa identities with those of other known α-CoVs. The structural proteins E, M, and N of AcCoV-JC34 showed differences compared to homologues of known CoVs. The most conserved M protein had 46.3 to 92.3% sequence identity relative to those of α-CoVs. The N protein was most variable with only 21.3 to 77.4% sequence identity compared to those of α-CoVs at the aa-sequence level. Homologues of the ORF3, ORF6, ORF8, and ORF9 proteins could be found among some CoVs but with low similarity. Previous studies have shown that the ORF3, ORF6, and ORF9 proteins of CoVs may play different functions for the viral life cycle and pathogenesis, although more studies are needed to discern the functions of the NS proteins of AcCoV-JC34.

### Phylogenetic features of rodent CoVs

The first phylogenetic tree was constructed based on 400-bp RdRp sequences. In this tree, JC54 and JC34 clustered in the α-CoVs, within rodent and shrew CoVs (Fig. 1). JC34 was distantly related to the branch formed by the closely related CoV strains JC54, UKRn1, and LNRV (Lucheng-19). The other 21 CoV sequences detected from *A. chevrieri* or *A. ilex* clustered in β-CoVs and formed an independent lineage together with HKU24 from *R. norvegicus* and Longquan-353 from *A. agrarius*, in China. The 20 sequences detected from *A. chevrieri* were further divided into two branches.

**Fig. 3:**
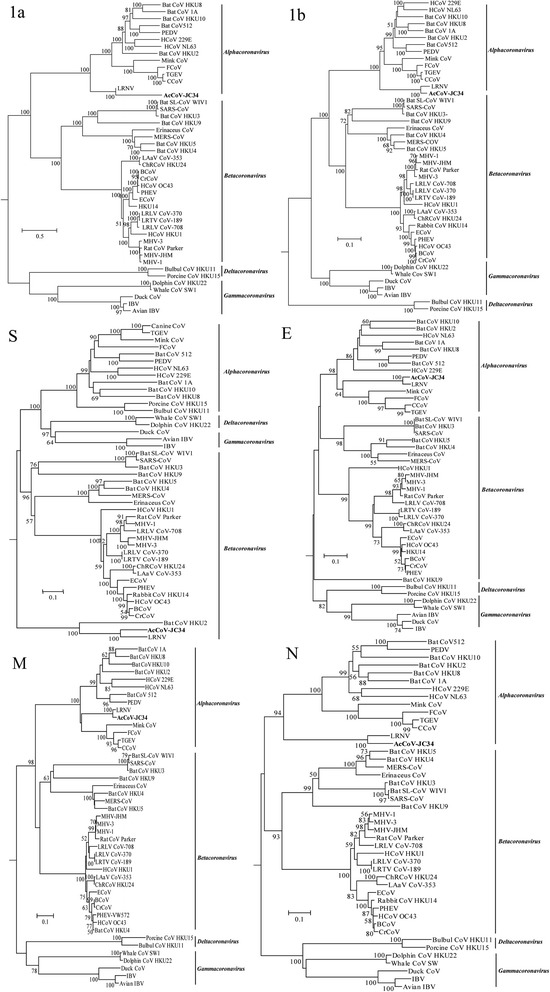
Phylogenetic analyses of AcCoV-JC34 based on amino acid sequences of ORF1a, 1b, S, E, M, and N. The trees were constructed by the maximum-likelihood method with 1000 bootstrap replicates. Bootstrap values above 50% are shown. AcCoV-JC34 identified in this study is shown in bold. The abbreviations and GenBank accession numbers are the same as those used in Fig. 1

Using predicted protein sequences, we further analyzed the phylogenetic features of AcCoV-JC34. In the phylogenetic trees constructed based on polyprotein 1a and 1b, AcCoV-JC34 clustered in the same branch with a rat CoV LRNV. Interestingly, in the tree based on the S protein, AcCoV-JC34 clustered with a rat CoV LRNV and a bat CoV HKU2 and formed a branch that appeared distinct from α-CoVs, β-CoVs, γ-CoVs, and δ-CoVs (Fig. 3). In the trees based on other genes, AcCoV-JC34 and LRNV together formed independent branches. These results indicated that AcCoV-JC34 possessed a special evolutionary position and may have a common origin with LRNV and HKU2 for the S protein (Fig. 3).

## Discussion

We detected CoVs in three different rodent species (*A. chevrieri*, *A. ilex*, and *E. fidelis*) from the Yunnan Province of China. In this study, we found a much higher (21.4%) detection rate of CoV nucleic acids in *A. chevrieri* than detected previously in other rodent species (<5%) [19, 20]. In addition, both α-CoV and β-CoV were found in *A. chevrieri*, suggesting that *A. chevrieri* may play an important role as a natural CoV host. *A. chevrieri* is known as Chevrier’s field mouse and is a dominant and endemic species in southwest China [32, 33]. In the Yunnan Province, *A. chevrieri* is an important pest in agriculture and human diseases that has been identified as a natural reservoir for plague bacilli and hantavirus [33]. The detection of both α-CoV and β-CoV in *A. chevrieri* with high infection rates highlighted the importance of viral surveillance in *A. chevrieri* in the Yunnan Province, which may be helpful for disease prevention and control.

We further characterized a full-length genome of a novel α-CoV, AcCoV-JC34, from *A. chevrieri*. In all five conserved replicase domains, AcCoV-JC34 was the most closely related to a *R. norvegicus* CoV LNRV, but diverged from other α-CoVs, indicating that AcCoV-JC34 and LNRV belong to a novel α-CoV species. To our knowledge, AcCoV-JC34 is one of the few rodent α-CoVs with a complete genome.

The genome of AcCoV-JC34 had some unique features compared to other CoVs, such as a shorter nsp5 (3CL^pro^) and three variant TRSs. These sequences containing the genes or elements were verified by PCR and NGS. Analysis of the aa sequence showed that the proteins encoded by AcCoV-JC34 had very low similarities to other α-CoVs. In particular, the S protein sequence had <20% sequence identity to other α-CoVs (except for LNRV and HKU2), but had a little higher identity (20.6 to 22.1%) compared to β-CoVs. In addition, the N proteins normally were conserved among each of CoV genera, but the N protein of AcCoV-JC34 only shared approximately 25% aa sequence identity with other α-CoVs (except for LNRV) (Table 2). The phylogenetic trees for ORF1a, 1b, and N showed that AcCoV-JC34 and LNRV formed a distinct branch within but at the root position of α-CoVs, suggesting that AcCoV-JC34 and LNRV may represent a special evolutionary position among α-CoVs. More interestingly, in the phylogenetic trees of S, AcCoV-JC34, LNRV, and HKU2 formed a root branch including all CoVs. These results suggested that other unknown CoVs exist in rodents in nature. Further studies should be continued to reveal the prevalence, diversity, and evolution of rodent CoVs.

All samples used in this study were from rodent intestines, suggesting a possible enteric tropism of rodent CoVs. During previous decades of research, different tissue tropisms of rodent CoVs have been observed. As the prototype of rodent CoVs, different MHV strains can infect variant tissues, and the A59 strain is primarily hepatotropic, but the JHM strain is neurotropic [15–18]. RCoV and a strain of sialodacryoadenitis virus (SDAV) both primarily infect the respiratory tract [34]. However, the tropisms of recently identified rodent CoVs from China and Europe have not been confirmed. A CoV in lineage A of β-CoV detected in the alimentary tract samples of Norway rats, HKU24, probably has enteric tropism [20]. Another cluster of α-CoVs (PLMg1, UKMa2, UKMa1, and UKRn1) were only detected in liver samples of Norway rats, the bank vole, the wood mouse, and the noncyclic field vole, suggesting that they are hepatotropic [21]. Additional research identified rodent α-CoV LRNV and β-CoVs LAMV and LRLV, which came from diverse tissue types that made it difficult to predict the tissue tropism of these viruses [19]. Nonetheless, the extensively studied rodent CoVs (MHV and RCoV) could lead to severe or mild diseases in their hosts. Further studies are needed to determine the potential pathogenicity of AcCoV-JC34 along with other recently detected rodent CoVs.

In the AcCoV-JC34 genome, a predicted ORF3 protein (214 aa) was located between the S and E genes. The ORF3 protein of AcCoV-JC34 possessed 30 to 78% aa sequence identity with the homologous proteins encoded by other α-CoVs. This protein has variant names in different CoVs and was named ORF4 protein in human coronavirus 229E [35], non-structural protein 3 in human coronavirus NL63 [36], 3c-like protein or non-structural protein 3c in ferret coronavirus, 3c protein in feline coronavirus [37], 3b protein in transmissible gastroenteritis virus (TGEV) [38], and ORF3 protein in porcine epidemic diarrhea virus (PEDV) [39]. Normally, the ORF3 protein was considered as an accessory non-structural protein, but several studies showed that the ORF3 protein was a membrane protein related to virulence [35, 37, 38, 40]. However, with low similarities between the ORF3 of AcCoV-JC34 and previously studied proteins, more experiments are needed to understand its function.

The S protein of CoVs is responsible for receptor recognition, binding, and membrane fusion, and serves as the first key factor of host restriction by meditating viral entry. In different CoVs, the RBD can be located at the NTD or CTD in S1. For example, among the α-CoVs, CTD was characterized as RBD in HCoV NL63 (aa 476–616), 229E (aa 417–547), and PEDV [41–44], but the NTD was characterized as an RBD in the TGEV [45]. Here, the S1 of AcCoV-JC34 shared <20% aa sequence identity with those of very well characterized α-CoVs, which made it difficult to predict whether the RBD was located in NTD or CTD and which host molecule could be the possible receptor for AcCoV-JC34. The S2 of AcCoV-JC34 showed 40 to 50% identity to that of β-CoVs. By sequence alignment and SWISS-MODEL analysis (data not shown), we deduced the precise positions of FH, HR1, and HR2. The higher similarities between S2 of AcCoV-JC34 (HKU2) and β-CoVs than that to α-CoVs suggested that the structure and functional mechanism of S2 of AcCoV-JC34 may more homologous to β-CoVs.

Emerging infectious diseases caused by CoV are mostly due to interspecies transmission from animals to humans. Previous data indicated that bats are natural reservoirs for α- and β-CoVs [46]. A number of human CoVs, including SARS-CoV, MERS-CoV, HCoV229E, and NL63 might have originated from bats [47, 48]. Among the rodent CoVs, the receptor usage, tissue tropism, and pathogenesis of MHV have been studied in detail [49]. However, novel CoVs, like AcCoV-JC34, HKU24, LRNV, LAMV, and LRLV are not fully understood. Identification of the receptor for these viruses could help in evaluating the potential host range and ability for interspecies transmission from rodents to other mammals. Although most of these novel rodent CoVs have been characterized with full-length or near full-length genome sequences, the lack of successfully isolating those viruses thoroughly restricts future studies. More positive samples and cell lines will facilitate viral culture in the future. In addition, more attention should be paid to the diversity of CoVs in rodent, which could help to better understand the role of rodents in the evolution and ecology of CoVs.

## Conclusions

The results of this study revealed that diverse α-CoVs and β-CoVs are circulating in rodents in the Yunnan Province of China and highlighted the importance of rodents as a natural reservoir for CoVs. The complete genome of Ac-JC34 with new characteristics and a special S gene provided new insights into the genetics and evolution of CoVs. These findings should be useful for future genomic studies of CoVs and for further functional studies of S proteins.

## Additional file

Additional file 1: Figure S1. Geographical map of Jianchuan country and the sampling areas. **Figure S2.** Alignment and predicted domains and cleavage sites of AcCoV-JC34 spike protein. NTD, N-terminal domain; RBD, receptor-binding domain; HR, heptad repeat; TM, transmembrane anchor. The signal peptide corresponds to residues 1 to 19. The cleavage sites were indicated by arrows. (PDF 223 kb)

## Abbreviations

*A. chevrieri*: *Apodemus chevrieri*
*A. ilex*: *Apodemus ilex*
aa: Amino acid
CoVs: Coronaviruses
CTD: C-terminal domain
CytB,: Cytochrome b
E: Envelope
*E. fidelis*: *Eothenomys fidelis*
FP: Fusion peptide
HR: Heptad repeats
M: Membrane
MERS-CoV: Middle East respiratory syndrome coronavirus
MHV: Mouse hepatitis virus
N: Nucleocapsid
NGS: Next-generation sequencing
nt: Nucleotide
NTD: N-terminal domain
ORFs: Open reading frames
PDB: Protein Data Bank
PEDV: Porcine epidemic diarrhea virus
RBD: Receptor-binding domain
RdRp: RNA-dependent RNA polymerase gene
RT-PCR: Reverse transcription PCR
S: Spike
SARS-CoV: Severe acute respiratory syndrome coronavirus
SDAV: Sialodacryoadenitis virus
TGEV: Transmissible gastroenteritis virus
TRS: Transcription regulatory sequence
α-CoV: *Alphacoronavirus*
β-CoV: *Betacoronavirus*
γ-CoV: *Gammacoronavirus*
δ-CoV: *Deltacoronavirus*
