# Supplementary material for: Detection of alpha- and betacoronaviruses in rodents from Yunnan, China
Source: Virol J. 2017 May 26;14:98. doi: 10.1186/s12985-017-0766-9 (PMC5446729; doi:10.1186/s12985-017-0766-9)
Supplement: Additional file 1: Figure S1. — Geographical map of Jianchuan country and the sampling areas. Figure S2. Alignment and predicted domains and cleavage sites of AcCoV-JC34 spike protein. NTD, N-terminal domain; RBD, receptor-binding domain; HR, heptad repeat; TM, transmembrane anchor. The signal peptide corresponds to residues 1 to 19. The cleavage sites were indicated by arrows. (PDF 223 kb) [file 12985_2017_766_MOESM1_ESM.pdf]

Supplemental Materials:

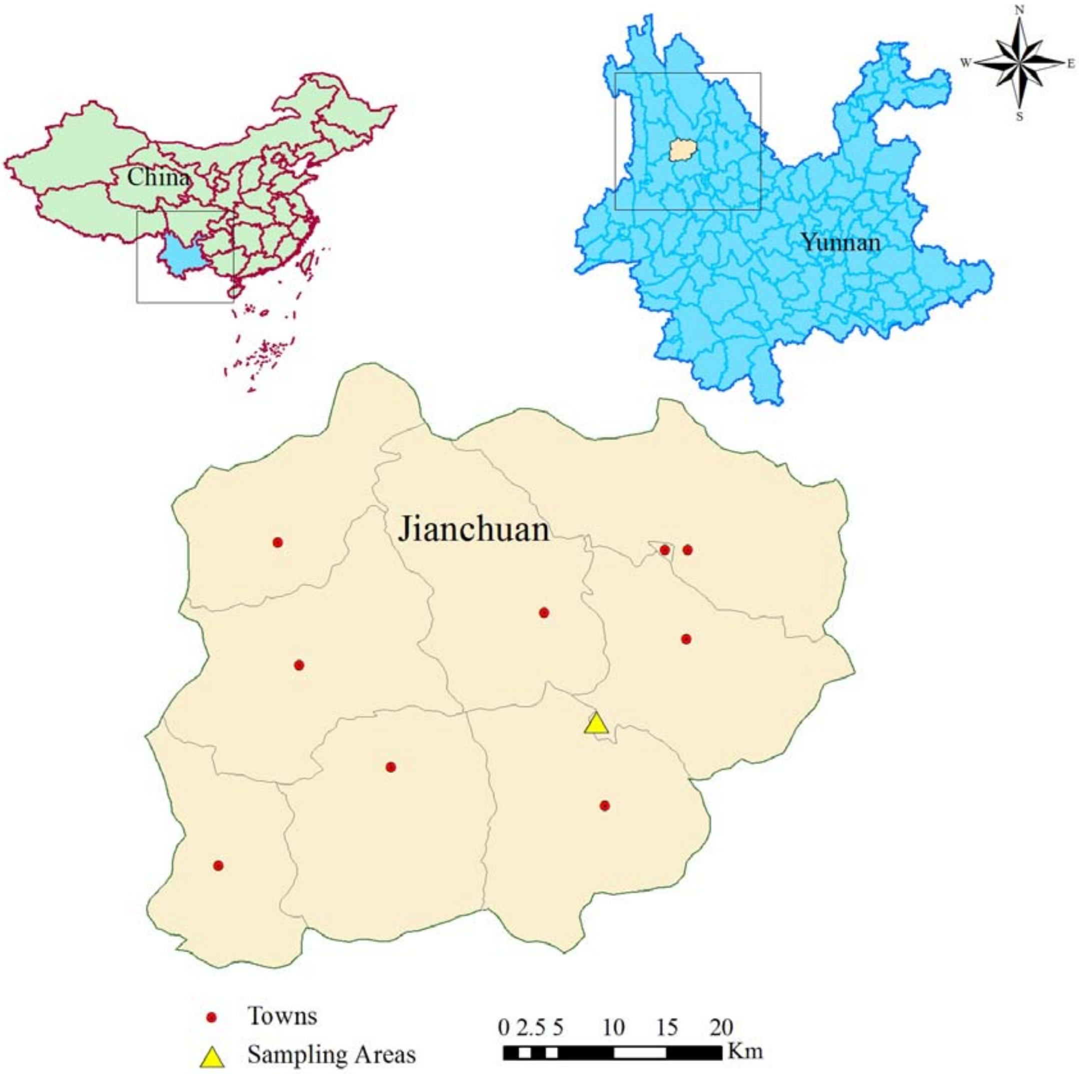

Fig S1. Geographical map of Jianchuan country and the sampling areas.

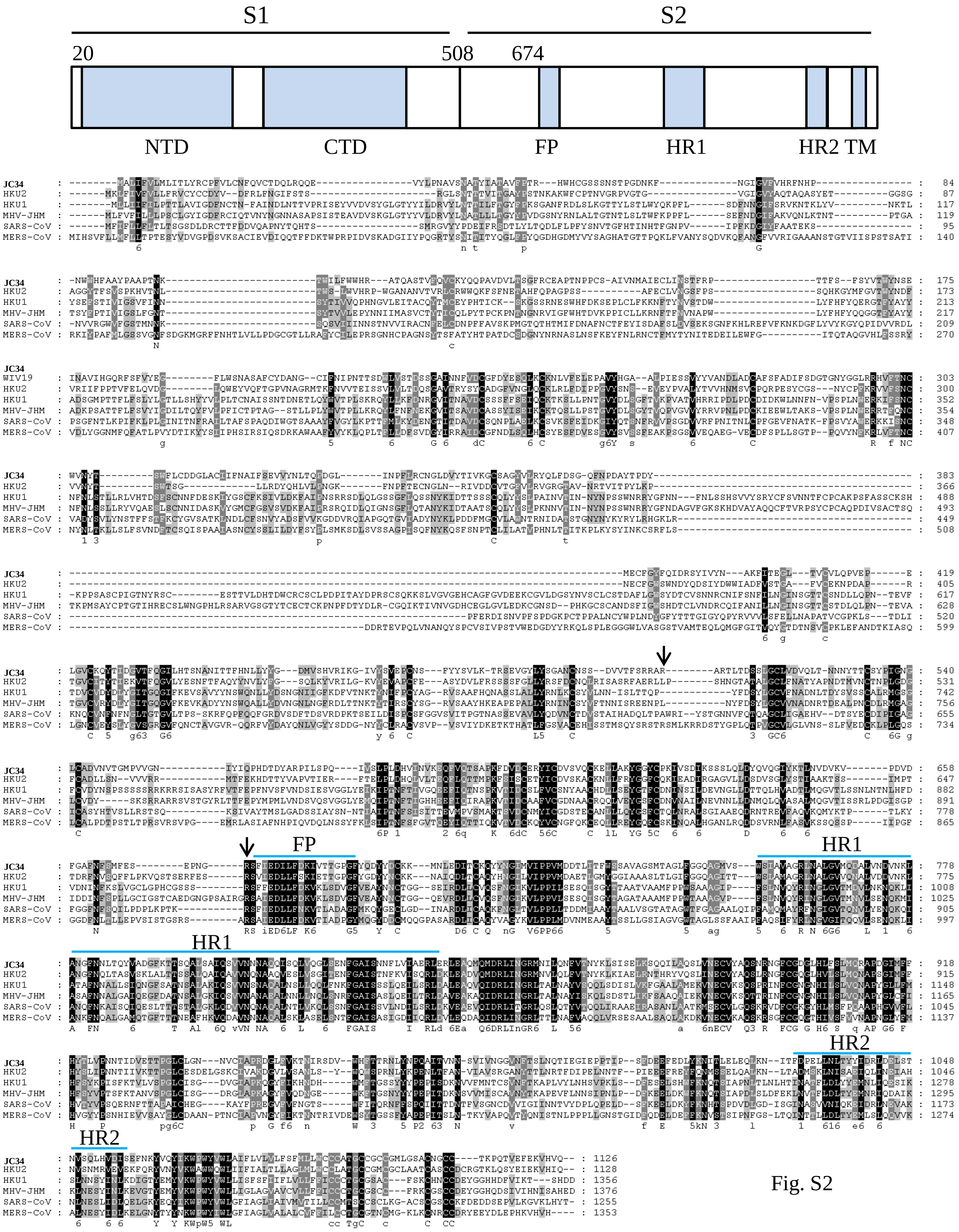
S1
S2
20
508
674
NTD
CTD
FP
HR1
HR2 TM
JC34
WIV19
HKU2
HKU1
MHV-JHM
SARS-CoV
MERS-CoV
FP
HR1
HR2

Fig. S2

Fig S2. Alignment and predicted domains and cleavage sites of AcCoV-JC34 spike protein. NTD, N-terminal domain; RBD, receptor-binding domain; HR, heptad repeat; TM, transmembrane anchor. The signal peptide corresponds to residues 1 to 19. The cleavage sites were indicated by arrows.
